# An online browser-based attentional blink replication using visual objects

**DOI:** 10.1371/journal.pone.0289623

**Published:** 2023-08-03

**Authors:** Deena Sharabas, Manuel Varlet, Tijl Grootswagers

**Affiliations:** 1 The MARCS Institute for Brain, Behaviour and Development, Western Sydney University, Sydney, Australia; 2 School of Psychology, Western Sydney University, Sydney, Australia; Groningen University, NETHERLANDS

## Abstract

The complex relationship between attention and visual perception can be exemplified and investigated through the Attentional Blink. The attentional blink is characterised by impaired attention to the second of two target stimuli, when both occur within 200 – 500ms. The attentional blink has been well studied in experimental lab settings. However, despite the rise of online methods for behavioural research, their suitability for studying the attentional blink has not been fully addressed yet, the main concern being the lack of control and timing variability for stimulus presentation. Here, we investigated the suitability of online testing for studying the attentional blink with visual objects. Our results show a clear attentional blink effect between 200 to 400ms following the distractor including a Lag 1 sparing effect in line with previous research despite significant inter-subject and timing variability. This work demonstrates the suitability of online methods for studying the attentional blink with visual objects, opening new avenues to explore its underlying processes.

## Introduction

The sensory information in our visual field is constantly changing. As a result, the visual system must adapt to the overwhelming number of stimuli it is presented with. Attentional mechanisms filter irrelevant stimuli and select inputs that require further processing [1–3]. These selection mechanisms are highlighted in the attentional blink [4, 5], a robust effect where a salient event in a stream of stimuli disrupts the processing of subsequent stimuli for a short duration (200-500ms) [5]. A target stimulus presented in this time window is often missed. The attentional blink has been subjected to much debate around its theoretical origins [1, 6–11], which even after decades of research remain an open question. The recent increase in popularity of online behavioural testing for its fast and scalable benefits [12] may help shed new light on phenomena such as the attentional blink. However, when moving towards large-scale online behavioural testing instead of classic controlled experimental lab settings, it is important to establish and measure attentional blink in paradigms that are well suited for online experiments.

The attentional blink has been well studied in experimental lab settings [13–16], and a growing number of studies have now been able to successfully replicate the attentional blink in online behavioural experiments [17, 18]. However, online experimental settings continue to bring challenging issues for attentional blink research as it relies on fast and accurate stimulus presentation timings [12]. While previous online replications used a classic letter-based two-target attentional blink design, the suitability of online methods for a picture-based attentional blink has not been fully established [2, 19, 20]. This is important because the relatively quick hypothesis-testing in large samples enabled by online testing will allow the development of new theoretical insights into the attentional blink.

The current study attempts to reproduce a picture-based attentional blink effect in a short and simple online study. The study follows a simplified distractor and target design, with random pattern masks as filler stimuli. With this simplified design we aimed to isolate the attentional effects of the distractor and its subsequent impact on target identification accuracy, while minimising the size of the experiment which is important for running in an online environment. We further investigated variability on stimulus presentation timing accuracy and between-subject variability, which are two major concerns that arise from online testing methods. We hope our results and corresponding experiment code may help drive future research into the attentional blink.

## Methods

All materials and data are available online at https://osf.io/atjpe/.

### Participants

100 undergraduate psychology students (18–51 years, *M*: 22.3, *SD*: 6.6) participated in the experiment in exchange for course credit. Each participant was to self-assess their vision as normal (or corrected-to-normal) to participate in the study. The experiments were approved by the Western Sydney University Human Research Ethics Committee. Participants provided informed written consent and were free to withdraw from the study without consequence. After the study, they were debriefed about the aims of the study. Participant data was analysed anonymously.

### Design and procedure

**Fig 1:**
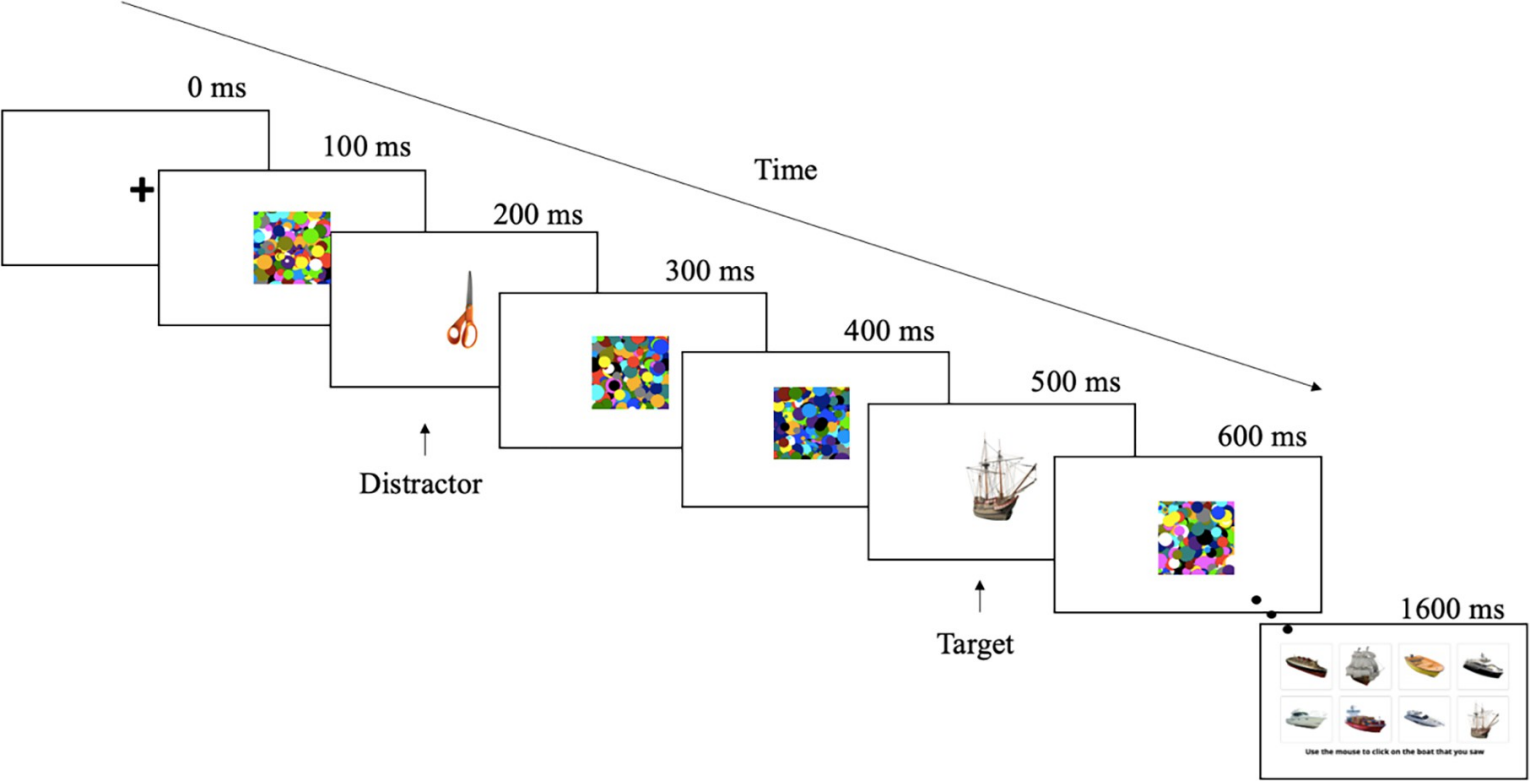
Illustration of a typical trial. Images were shown in an RSVP sequence for 100ms each. Here, the target stimulus is positioned at Lag 3. At the end of each sequence, participants were required to select the specific boat image that was presented.

The study was conducted online [12], the only requirement for participation was a keyboard and mouse. The experiment was programmed in Javascript (adapted from [21]) using JsPsych version 6.1.0 [22] and was hosted on the pavlovia platform [23]. The experiment ran on participant’s own computer with the only criterion that the size of the display was at least 600x400 pixels. The experiment was based on a 4 (Lag 1,2, 3 and 4) x 2 (Distractor and Control) within-subject design to replicate the classic attentional blink paradigm [4, 5, 7]. Stimuli were presented in an RSVP stream of 16 random dot images [24], and including a target (one of 8 boats) and a distractor placed within 1–4 positions before the target. Distractor stimuli were selected randomly out of 200 objects. Stimuli were obtained from [21, 25]. A control condition included no distractor stimulus and only a target. The participant’s task was to select the boat image that was shown as part of the RSVP sequence (Fig 1). The next trial started after they clicked on any of the boat images. Participants received no feedback on their accuracy and we did not exclude participants based on their accuracy.

Response accuracy was recorded after each trial, with 384 trials total. There were an equal number of trials for each level of Lag, with 88 trials per Lag. 32 trials were control trials with no distractor. All trials were presented in random order. We did not analyse reaction times. The trial began with a blank, white background and a fixation cross in the centre (Fig 1). Stimuli occupied 225 pixels of the participant’s display monitor, resulting in a visual angle of approximately 4° (based on a 13-inch monitor at a 50 cm distance). The duration of each trial was about 1.6 s, with a presentation rate of 100 ms per stimulus. The experiment lasted approximately 40 minutes, with a loading bar at the top of the monitor indicating the participant’s progression.

## Results

Our aim was to replicate the attentional blink effect online using a minimalistic paradigm with Mondrian masks as filler stimuli and visual objects as both targets and distractors. We presented distractor stimuli at Lags 1 to 4, and measured target identification accuracy (the percentage of trials where the correct boat image was selected) between distractor present and distractor absent trials.

### Attentional blink effect

**Fig 2:**
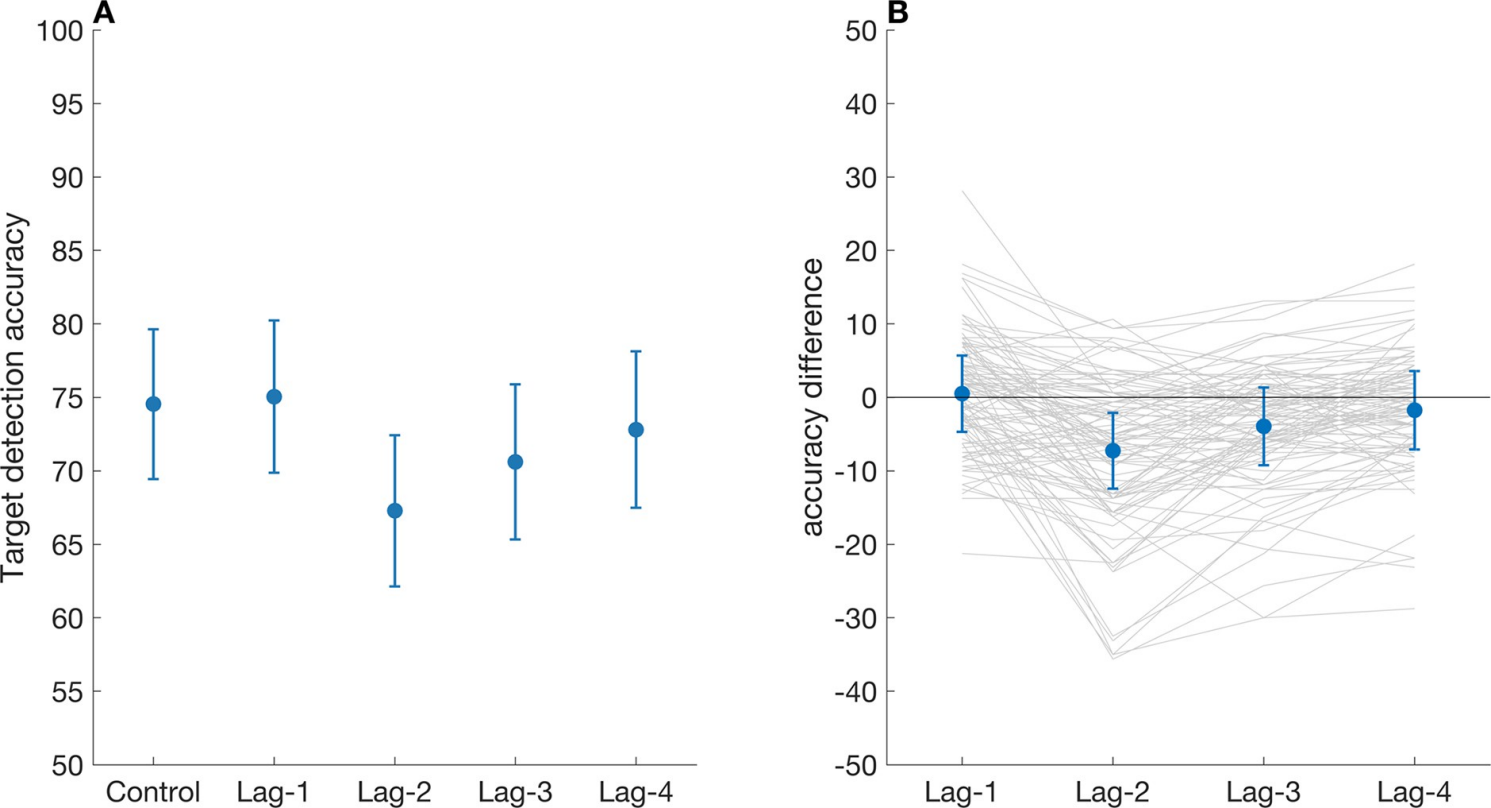
**A.** Response accuracy as a function of attentional blindness through distractors at various Lag placements. The control condition had just a target and no distractor. Displayed are the mean and the within-subject 95% confidence interval. Here, a clear attentional blink effect is visible, with decreased target classification accuracy at Lag 2 and 3. **B.** The within-subject differences (mean & 95% confidence interval) between control and different Lags highlight the attentional blink effect observed at Lag 2 and 3.

The 4 x 2 ANOVA on response accuracy indicated a main effect for Lag (*F* [2.91, 296.95] = 10.72, *p* < .001, η^2^_g_ = 0.005), showing that response accuracy varied between Lag levels. The ANOVA also indicated a main effect of Distractor (*F* [1, 102] = 20.88, *p* < .001, η^2^_g_ = 0.003) showing that the presence of a distractor significantly reduced response accuracy. Finally, the ANOVA revealed a significant interaction between the factors Distractor and Lag (*F* [2.85, 290.72] = 4.27, *p* < .01, η^2^_g_ = 0.002), as illustrated in Fig 2.

Post-hoc pairwise comparisons with a Bonferroni correction indicated significant differences (p < .05) between all pairs except for Lag1-control, Lag1-Lag4, and Lag4-control. The attentional blink effects were only established when the distractor was at Lag 2 or Lag 3, and there was no evidence for a difference in target accuracy between Lag 1, Lag 4 and the no-distractor (control) condition (all ps > .05). These results highlight a clear attentional blink effect at Lag 2, as well as a small carry-over effect at Lag 3.

### Reliability of online presentation timing

**Fig 3:**
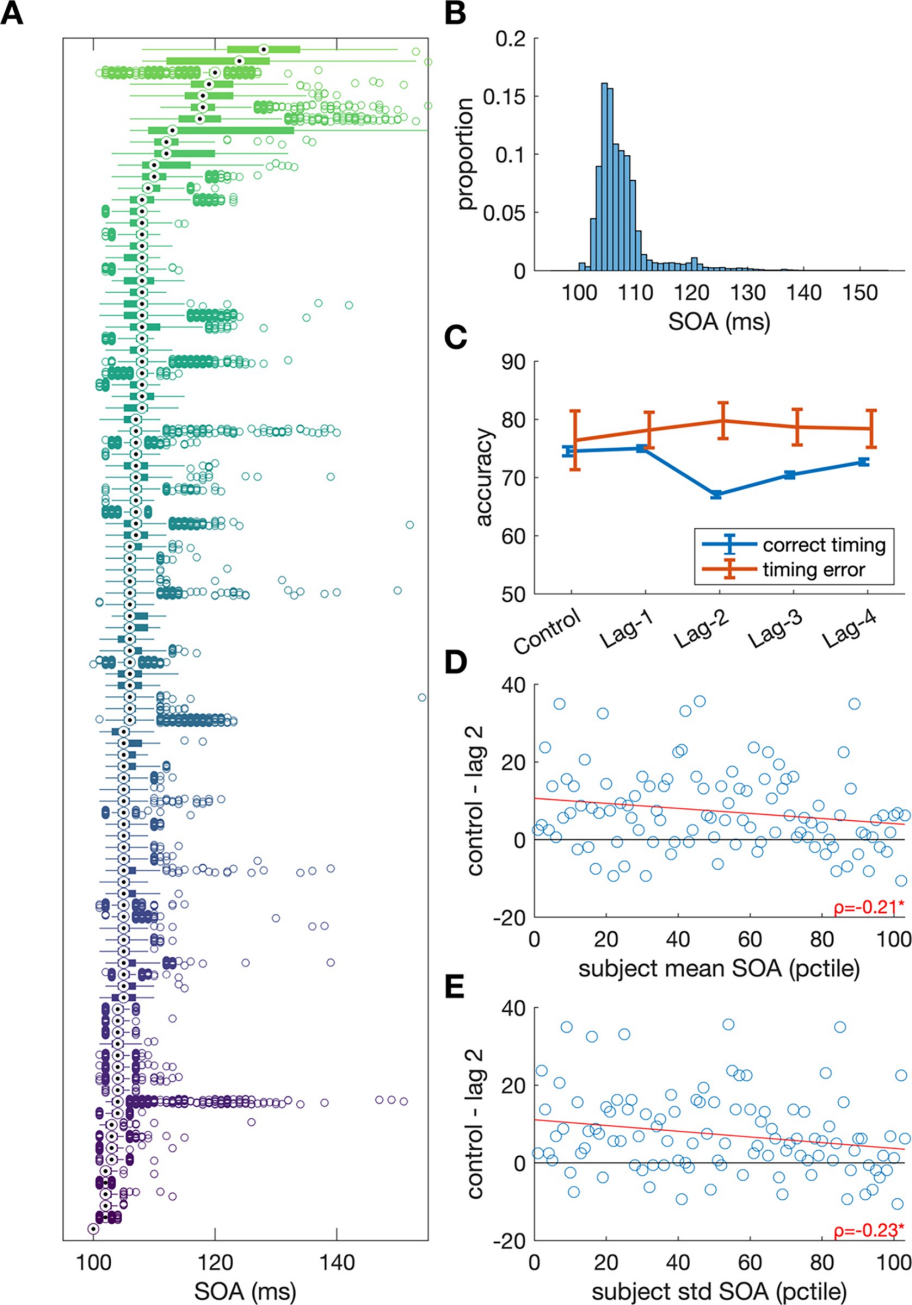
Presentation timing results. Stimuli were requested to be presented at 100 ms intervals. **A.** Distributions of presentation timings across the whole experiment for each participant (medians, quartiles, and outliers) show precise timings for most subjects. **B.** Histogram of all presentation timings showing that the vast majority is within one screen refresh. **C**. Comparison of results using only accurate timing trials (approx. 8050 trials in each Lag and approx. 3224 trials in the control condition) with trials that had at least one timing error (approx. 180 trials in each Lag and 72 trials in the control condition). Note the large standard errors due to the relatively few trials with timing errors. **D&E.** Weak negative correlation between the attentional blink effect (i.e. the accuracy difference between Lag 2 and control) and mean SOA (D) or standard deviation of the SOA (E).

As online experiments do not allow any control over the experiment setup, we asked how reliable the timing was. Fig 3 shows the stimulus timing as reported by the participant’s browsers. For most subjects (about 75%), timing was highly accurately within one screen refresh of 100 ms (Fig 3A & 3B). When analysing separately the trials with timing errors, the attentional blink effect disappeared (Fig 3C). However, even for the subjects with larger timing fluctuations (about 25%), the results (Fig 3D & 3E) suggest that most showed the attentional blink effect (where accuracy in the control was larger than in the Lag-2 condition). Still, there was weak evidence for a correlation between attentional blink effect size (i.e., the difference between Lag 2 and control) and the i) mean (Fig 3D) and ii) standard deviation (Fig 3E) of stimulus onset asynchrony (SOA).

## Discussion

The present study aimed to establish the replicability of the attentional blink with visual objects in online settings. Our results showed a clear attentional blink effect, with a significant decline in response accuracy when a distractor is present [4, 26, 27]. The experiment also replicated the temporal progression of attentional decline that has been demonstrated in previous attentional blink studies [5]. With peak response accuracy at Lag 1, a sharp attentional decline at Lag 2, followed by a gradual increase in response accuracy in subsequent Lags. Interestingly, the significant interaction effect in response accuracy at various Lag and distractor combinations, reproduced within our unconventional distractor/Mondrian mask paradigm, reinforces the replicability of the attentional blink. As expected, the non-significant difference in response accuracy at Lag 1 supports the Lag 1 sparing effect, that is, the inclusion of a distractor has no effect on response accuracy at Lag 1.

It is difficult to compare our results to existing lab-based attentional blink work, as our design is unusual compared to standard attentional blink paradigms which are generally using letters and two targets, rather than a target/distractor design. For a more direct comparison into the online replication of more standard attentional blink paradigms, see previous work [e.g., 17, 18]. Our design has similarities to picture based designs such as the emotional blink paradimgs [28–30], with the main difference being our use of Mondrian masks as fillers. As our paradigm generates a reliable attentional blink effect, it would be well suited for experiments that attempt to isolate the influence of particular image properties (e.g., emotional content) on attention.

Online experiments are ideally suited for exploring these directions further, as they facilitate data collection from large populations. They can be conducted with a large number of participants (n>100) with relatively little effort whereas recruiting and running 100 participants in a lab-based study is often a time-consuming and expensive process. This is therefore a promising methodological approach to conduct multiple highly powered behavioural experiments in a relatively short time and progress the field faster.

Exploratory investigations in the stimulus timing results showed generally highly accurate timing (for about 75% of participants, as reported by the browser), which highlights the reliability of browser-based experiments. Past work has already shown accurate timing for online experiments [17, 18], but here we show this also holds when more demanding stimuli (pictures) are displayed in fast succession. Furthermore, the strength of the attentional blink effects does not seem to be directly affected by increased variability in stimulus presentation timing. Similar effects were observed in participants who had greater stimulus presentation timing issues (about 25% of participants). However, the large timing variability suggest that online studies should ideally aim to use within-subject manipulations, and reliable between-subject effects may be harder to obtain due to the large variability in timing, which may exacerbate the between-subject variability in attentional blink effects [e.g., 31]. Future studies could use the browser-reported timing precision to exclude participants.

To conclude, this study demonstrates the suitability of online attentional blink paradigms with visual objects. Reliable attentional blink effects can be obtained with visual objects online despite stimulus presentation timing issues that might arise in browser-based experiments. This opens new avenues for studying the attentional blink and its underlying processes in large scale online studies.
